# Thyroid Function and Body Weight: A Community-Based Longitudinal Study

**DOI:** 10.1371/journal.pone.0093515

**Published:** 2014-04-11

**Authors:** Lena Bjergved, Torben Jørgensen, Hans Perrild, Peter Laurberg, Anne Krejbjerg, Lars Ovesen, Lone Banke Rasmussen, Nils Knudsen

**Affiliations:** 1 Research Centre for Prevention and Health, The Capital Region of Denmark, Glostrup, Denmark; 2 Department of Endocrinology, Bispebjerg University Hospital, Copenhagen, Denmark; 3 Faculty of Health Sciences, Copenhagen, Denmark; 4 Faculty of Medicine, Aalborg University, Aalborg, Denmark; 5 Department of Endocrinology, Aalborg University Hospital, Aalborg, Denmark; 6 Department of Gastroenterology, Slagelse Hospital, Slagelse, Denmark; 7 Department of Nutrition, National Food Institute, Technical University of Denmark, Søborg, Denmark; University of Bristol, United Kingdom

## Abstract

**Objective:**

Body weight and overt thyroid dysfunction are associated. Cross-sectional population-based studies have repeatedly found that thyroid hormone levels, even within the normal reference range, might be associated with body weight. However, for longitudinal data, the association is less clear. Thus, we tested the association between serum thyrotropin (TSH) and body weight in a community-based sample of adult persons followed for 11 years.

**Methods:**

A random sample of 4,649 persons aged 18–65 years from a general population participated in the DanThyr study in 1997–8. We included 2,102 individuals who participated at 11-year follow-up, without current or former treatment for thyroid disease and with measurements of TSH and weight at both examinations. Multiple linear regression models were used, stratified by sex and adjusted for age, smoking status, and leisure time physical activity.

**Results:**

Baseline TSH concentration was not associated with change in weight (women, P = 0.17; men, P = 0.72), and baseline body mass index (BMI) was not associated with change in TSH (women, P = 0.21; men, P = 0.85). Change in serum TSH and change in weight were significantly associated in both sexes. Weight increased by 0.3 kg (95% confidence interval [CI] 0.1, 0.4, P = 0.005) in women and 0.8 kg (95% CI 0.1, 1.4, P = 0.02) in men for every one unit TSH (mU/L) increase.

**Conclusions:**

TSH levels were not a determinant of future weight changes, and BMI was not a determinant for TSH changes, but an association between weight change and TSH change was present.

## Introduction

The thyroid gland produces thyroid hormones, which regulate gene transcription and metabolism throughout the body. Thyroid hormones play a key role in the basal metabolic rate, thermogenesis, as well as in the regulation of body metabolism in starved and fed states [1]–[3]. Overt thyroid disease is associated with marked changes in energy expenditure and body weight, with enhanced protein breakdown, lipolysis [4], [5], and typically weight loss in hyperthyroidism, and the reverse in hypothyroidism [6]. Moreover, small changes in T4 dose in patients in long-term T4 treatment have been shown to modify resting energy expenditure significantly [7].

The main role of adipose tissues is to store energy to meet the energy needs of the body and to protect it from excess glucose by converting this to and storing it as triglycerides. Recently, adipose tissue has been recognized as a major endocrine organ, involved in the regulation of thermogenesis, food intake, and energy expenditure, e.g., the change between fed and starved state [3]. Studies of weight reduction by bariatric surgery [8] or gastric banding [9] have found postoperative changes in levels of TSH (serum thyrotropin), free T4 (thyroxine) and free T3 (triiodothyronine), but in different directions. Likewise, a decrease in TSH was observed following diet-, exercise-, and behavior therapy-induced weight loss in children and adolescents [10], [11]. Conversely, obesity induced by high-fat diet weight gain has a significant effect on T4 and T3 levels in rats [12]. A relationship between body weight and thyroid hormones seems conceivable. The exact mechanisms are as yet unexplained and the temporality is debated.

Evidence from several cross-sectional population-based studies has shown a significant positive association between body mass index (BMI) and serum TSH, even for values of TSH within the normal reference range [13]–[18], among those a study by Knudsen et al. on baseline data from this cohort [16]. However, the number of longitudinal studies of the association are limited [14], [17], [19], [20] and the results are conflicting. The aim of the present study was to investigate the association between 11-year longitudinal changes in thyroid function as assessed by serum TSH and changes in body weight in a large sample of adult Danes. Furthermore, we wished to test the hypothesis that the relationship between serum TSH and free T4 is dependent on weight; a relation previous suggested to be a useful measure of the abnormal thyroid hormone sensitivity in a study of patients with hereditary pituitary resistance to thyroid hormones [21].

## Materials and Methods

### Ethics Statement

Participants gave their written informed consent, and studies were approved by the North Denmark Region Committee on Health Research Ethics (Nos. 2-16-4-0001-97, VN 96/208mch, and N-VN-19960208mch), and the Danish Data Protection Agency. The recommendations of the Declaration of Helsinki were followed.

### Design and Population

The DanThyr program, described earlier in detail [22], is a Danish program monitoring the presence and development of thyroid disease before and after iodine fortification of household salt and salt used in the commercial production of bread since 1^st^ July 2000. The present study used data from C1 cohort in the DanThyr program initiated in 1997/98 (C1a, N = 4,649, 50.1% participation) [23] and followed up in 2008–2010 (C1b, N = 2,465, 59.1% of baseline participants) [24]. The random samples were drawn from Copenhagen in the eastern part of Denmark and Aalborg in the western part of Denmark, representing areas with pre-fortification mild and moderate iodine deficiency, respectively. Using the national Civil Registration system, in which all inhabitants of Denmark can be identified by a unique 10 digit number, the sampling included four age groups of women (18–65 years) and one age group of men (60–65 years).

Participants underwent physical examination, gave a non-fasting venous blood sample and a spot urine sample, and had a thyroid ultrasonography (US) performed (Siemens Sonoline Versa Pro, Siemens, Erlagen, Germany). All procedures (e.g., invitation, questionnaires, and examination) were similar at baseline and follow-up examination. Blood and urine samples were collected between 08.00 a.m. and 5.30 p.m., stored at −20°C, and subsequently analyzed in random order with respect to region, sex, age, and season of the year at examination, at baseline, and follow-up. The normal reference range was defined in the baseline cohort as follows: TSH, 0.4–3.6 mU/L; free T4, 9.8–20.4 pmol/L [23].

**Figure 1:**
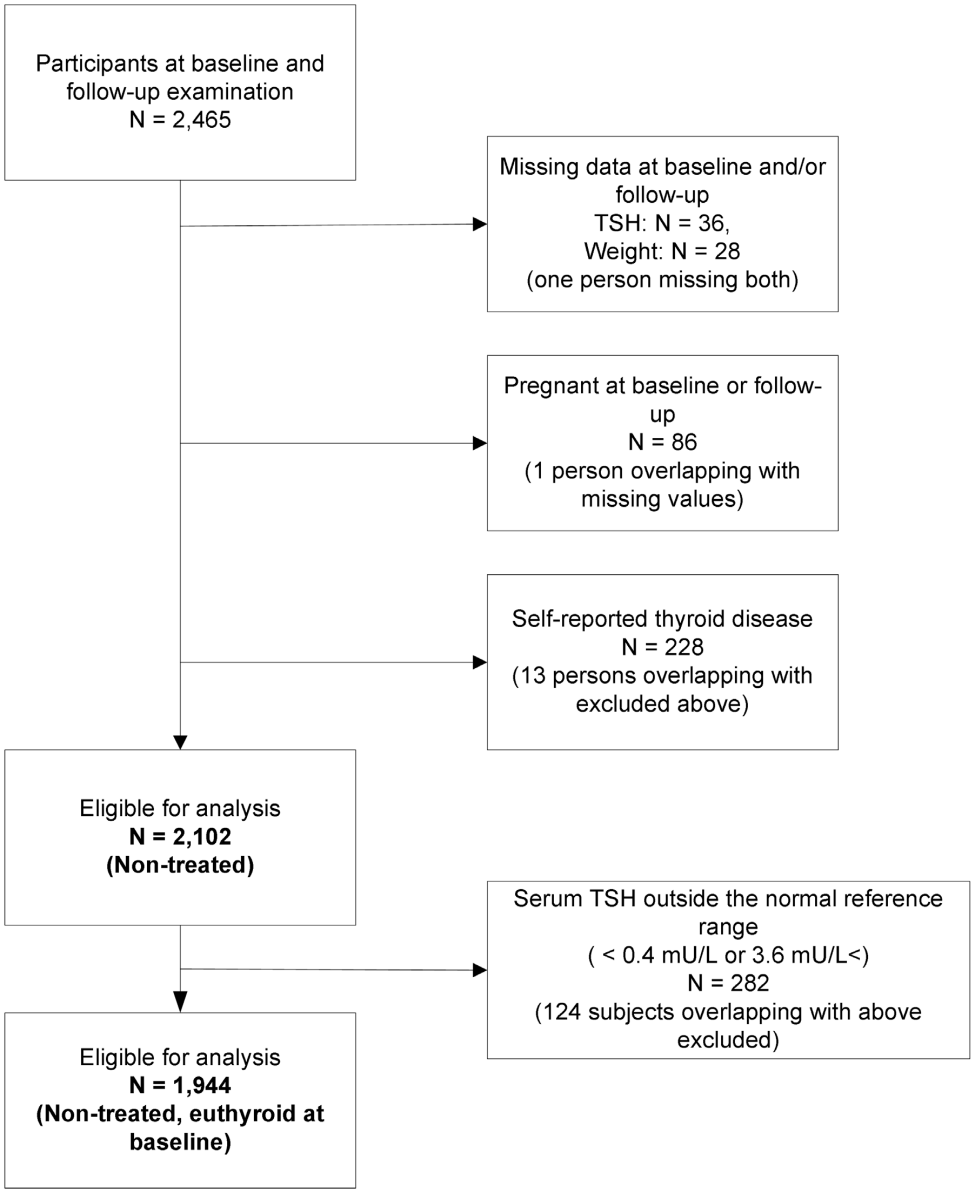
Flow chart of the study group. *TSH* serum thyrotropin.

Among the 2,465 examined at both baseline and follow-up, 36 had missing serum TSH values, 28 had missing measurement of weight, 86 persons were pregnant, 228 had current or former treatment for thyroid disease (medicine, surgery, or radioactive iodine therapy), leaving 2,102 participants for analysis (**Figure 1**). Moreover, we replicated all analysis after exclusion of 282 participants with a baseline serum TSH concentration outside the normal reference range (below 0.4 mU/L or above 3.6 mU/L), leaving 1,944 participants for analysis. They were defined as being euthyroid at baseline (shown in **Figure 1**). This was done to increase comparability with related studies.

### Analyses

LUMItest assays were used to measure TSH and free T4 at baseline (BRAHMS, Berlin, Germany) and the Roche Modular E® system by electrochemical luminescence using ELECSYS® (Roche Diagnostics 6M6H, Mannheim, Germany) at follow-up. The lower limit of detection was 0.01 mU/L at baseline and 0.014 mU/L at follow-up. The size of the bias induced by the change in assay from baseline to follow-up was calculated in a subsample of 201 blood samples. The Bland-Altman plot showed very low inter-assay variation without marked irregularities (data not shown). The bias for TSH was modest (mean difference: 0.019 mU/L, 95% confidence interval [CI] −0.25 to 0.29 mU/L). Reanalysis of serum free T4 showed higher values when analyzed with the follow-up assay (1.65 pmol/L, 1.30–2.00 pmol/L). TPO-Ab measurements were analysed by radioimmunoassay (RIA) technique by DYNOtest anti-TPO method (BRAHMS Diagnostica, Berlin, Germany) at baseline, as described earlier [25] and KRYPTOR anti-TPO_n_ (BRAHMS Diagnostica, Berlin, Germany), an automated antibody assays were used at follow-up [26]. No difference in CV performance between the two assays was found in a previous study [26]. Positive TPO-Ab status was defined as 30 kU/liter or higher according to the functional sensitivity given by the manufacturer and the limits used in previous DanThyr studies [25]. Urine iodine concentrations were determined at both examinations using the Ce/As method after digestion by alkaline ashing, as previously described [27], [28]. The analytical sensitivity of the assay was 2 µg/L. The iodine laboratory was certified by the U.S. Centers for Disease Control and Prevention EQUIP program.

### Variable Definition

BMI was calculated as weight (kg) divided by the square of height (m^2^). Weight and height were measured when the participants were wearing light clothes and no shoes. Treatment with medicine, surgery, or radioactive iodine therapy for thyroid disease, smoking status, and leisure time activity were self-reported. Smoking was classified as: never smokers, former smokers, occasional smokers, and daily smokers. According to the 11-year change in smoking status, participants were classified as: current smokers (N = 572), never smokers (N = 949), former smokers (N = 402), those who stopped smoking during follow-up (N = 381), and those who started smoking during follow-up (N = 98). A dichotomized smoking status (daily smoking, yes/no) was used to test the interaction with smoking in the three models. According to leisure time physical activity, participants were classified as mainly sedentary; light physical activity, 2–4 hours a week; light physical activity, more than 4 hours a week; or heavy physical activity, more than 4 hours a week. Eleven-year changes in physical activity were classified as less active, unchanged, or more active.

### Statistical Analysis

Data were analyzed using SAS, version 9.2 (SAS Institute Inc. Cary, NC, USA). Two-sided probability values <0.05 were considered significant. Non-normally distributed variables were log transformed to obtain a normal distribution. Descriptive characteristics of the participants presented as percent (total) or median (interquartile range, IQR) were compared with non-parametric statistics, Kruskal-Wallis test. We used multiple linear regression analysis to model the associations in the following three models: 1) the association between baseline serum TSH and change in weight during follow-up; 2) the association between change in serum TSH and change in weight during follow-up; and 3) the association between baseline BMI and change in serum TSH concentration during follow-up. Furthermore, we modeled linear regressions of: 1) the association between baseline free T4 and change in weight during follow-up; and 2) the association between change in free T4 and change in weight. Men and women were analyzed separately because of the age and sex composition of the population. All the models were adjusted for the baseline value of the outcome to adjust for regression to the mean and to allow changes in the exposure variables to be interpreted as effects on outcome changes from baseline to follow-up [29]. In addition, we adjusted for age at baseline (categorical), change in smoking status, and change in leisure time physical activity. Because the age range was only 5 years for men, the age variable was left out in their analysis. We also divided the exposure variables in the above described regression models in quartiles to test for a non-linear relationship between predictor and outcome.

In additional analysis, the relationship between Δ TSH (TSH concentration at follow-up minus TSH concentration at baseline, as outcome) and Δ free T4 concentration (as predictor) in three weight change groups (tertiles) was evaluated by linear regression analysis, adjusting for age group, change in smoking, and change in leisure time physical activity (and for the baseline value of serum TSH). We added an interaction term between Δ free T4 and weight change tertiles to test if the regression lines for the three weight groups were significantly different. Slopes of the three regression lines were identified from the same model. This analysis was restricted to women.

We tested the validity of all linear models by checking normality of residuals, homogeneity of residuals, and linearity of the relationship between response variable and predictors.

Wilcoxon Rank test was used to compare the independent measures of baseline characteristics of participants and non-participants at the re-examination.

## Results

Descriptive characteristics of the participants are shown in **Table 1**. The average follow-up time was 11.2 years (range, 10.1–12.8 years). Weight increased by 3.0 kg (95% CI, 2.7–3.3 kg) during follow-up; weight did not change significantly in men, 60–65 years at baseline (−0.2 kg, 95% CI, −0.9–0.4 kg), whereas it increased significantly by 3.7 kg (95% CI, 3.4–4.1 kg) in women (18–65 years at baseline). The median serum TSH increased significantly from the first to the second examination in the total population of individuals (from 1.30 (95% CI, 0.91–1.89) to 1.44 (95% CI, 0.99–2.05), P<0.01), as well as among men (from 1.24 (95% CI, 0.83–1.82) to 1.40 (95% CI, 0.94–2.02), P<0.01) and women (from 1.31 (95% CI, 0.92–1.90) to 1.45 (95% CI 1.00–2.06), P<0.01).

**Table 1:** Serum thyrotropin status and body mass index according to characteristics.

			Baseline		Follow-up	
Characteristics		All participants % (N)	Median (IQR)		Median (IQR)	
			Serum TSH (mU/L)	BMI (kg/m^2^)	Serum TSH (mU/L)	BMI (kg/m^2^)
Age (years)	18–20, women	19.7% (415)	1.42 (0.98, 1.98)	22.1 (20.4, 24.6)	1.46 (1.11, 2.00)	23.6 (21.2, 26.9)
	25–30, women	20.8% (436)	1.36 (0.99, 1.92)	22.4 (20.6, 24.3)	1.49 (1.04, 2.09)	23.5 (21.5, 26.6)
	40–45, women	26.9% (566)	1.23 (0.85, 1.78)	24.1 (21.9, 27.0)	1.42 (0.93, 2.07)	25.5 (23.0, 28.9)
	60–65, women	13.6% (286)	1.31 (0.84, 1.96)	26.2 (23.9, 29.0)	1.40 (0.94, 2.11)	26.6 (24.1, 29.7)
	60–65, men	19.0% (399)	1.24 (0.83, 1.82)	26.3 (24.6, 28.9)	1.40 (0.94, 2.02)	26.6 (24.9, 29.5)
	P values^a^		0.0019	<0.0001	0.39	<0.0001
Leisure time physical activity	Sedentary	11.4% (237)	1.22 (0.91, 1.92)	23.9 (21.5, 28.1)	1.40 (0.94, 2.09)	26.7 (22.9, 30.9)
	Light	45.9% (956)	1.28 (0.90, 1.82)	24.5 (21.9, 27.4)	1.41 (0.98, 2.00)	25.5 (22.8, 28.9)
	Moderate/vigorous	42.7% (889)	1.33 (0.92, 1.92)	23.9 (21.7, 26.3)	1.49 (1.02, 2.07)	24.8 (22.3, 27.3)
	P value^a^		0.34	0.009	0.24	<0.0001
Smoking status	Current smoker	32.3% (678)	1.21 (0.84, 1.76)	23.5 (21.2, 26.3)	1.22 (0.85, 1.78)	24.8 (22.1, 28.1)
	Never smoker	40.2% (846)	1.35 (0.96, 1.99)	24.0 (21.4, 26.8)	1.56 (1.10, 2.16)	25.1 (22.4, 28.2)
	Former smoker	19.6% (413)	1.30 (0.88, 1.97)	25.5 (23.2, 28.2)	1.43 (0.98, 2.08)	25.7 (23.3, 29.0)
	Occational smoker	7.8% (165)	1.28 (0.82, 1.84)	23.8 (22.1, 27.0)	1.43 (1.00, 1.98)	24.7 (22.3, 28.4)
	P value^a^		0.0003	<0.0001	<0.0001	0.0002
Region	Copenhagen	50.7% (1,065)	1.34 (0.92, 1.91)	24.1 (21.7, 27.1)	1.53 (1.06, 2.17)	25.0 (22.5, 28.2)
	Aalborg	49.3% (1,037)	1.27 (0.87, 1.82)	24.1 (21.8, 26.8)	1.35 (0.90, 1.92)	25.5 (22.7, 28.6)
	P value^a^		0.03	0.68	<0.0001	0.11
TPO-Ab status (kU/L)	≥30	21.1% (443)	1.42 (0.96, 2.12)	23.5 (21.7, 26.7)	1.78 (1.15, 2.80)	24.9 (22.8, 28.1)
	<30	78.9% (1,655)	1.28 (0.89, 1.82)	24.3 (21.8, 27.1)	1.39 (0.96, 1.91)	25.3 (22.6, 28.5)
	P value^a^		0.0001	0.057	<0.0001	0.52

a Kruskal-Wallis test.

*TSH* serum thyrotropin, *BMI* body mass index, *TPO-Ab* thyroid peroxidase antibody, *CI* confidence interval, *IQR* interquartile range.

The median serum TSH increased significantly from the first to the second examination in the total population of individuals (P<0.01), as well as among men (P<0.01) and women (P<0.01).

In the adjusted model, we found no association between baseline log TSH and weight change during follow-up in either sex among individuals without treatment for thyroid disease (women, P = 0.17; men, P = 0.72) and in persons with a serum TSH within the normal reference range at baseline (women, P = 0.19; men, P = 0.06). No association between baseline log BMI (modeled as a continuous variable) and TSH change was found, neither in all non-treated persons (women, P = 0.21; men, P = 0.85) nor in those with a baseline TSH concentration within the normal reference range (women, P = 0.78; men, P = 0.69).

A significant association between change in serum TSH concentrations and weight change was observed in both sexes (**Table 2**). In the adjusted model, weight increased by 0.3 kg (95% CI, 0.1–0.4) for every one-unit increment in serum TSH (mU/L) (P = 0.005) in women free of thyroid disease. In men, a one-unit increment in TSH was associated with a 0.8 kg (95% CI, 0.1–1.4, P = 0.02) increase in body weight. In the group of participants who had a baseline TSH within the normal reference range, weight increased by 0.6 kg (95% CI, 0.4–0.9, P<0.01) for every unit increment in serum TSH among women, and by 0.7 kg (95% CI, 0.02–1.3, P = 0.04) for every unit increment in TSH among men. Women with a serum TSH change in the lowest quartile had a mean weight increase of 1.2 kg (IQR −2.8, 5.4), whereas women in the highest quartile of change in TSH had a weight increase of 3.6 kg (IQR −0.2, 7.4) (P for trend<0.0001).

**Table 2:** Multiple linear regression of the prospective association between 11-year change in body weight and change in serum TSH in women and men.

		Number	Weight change per one-mU/L TSH increase	
			Model^a^ kg (95% CI)	Model^b^ kg (95% CI)
Individuals without thyroid disease	Women	1,703	0.2 (0.05–0.4)	0.3 (0.1–0.4)
			P = 0.01	P = 0.005
	Men	399	0.8 (0.9–1.4)	0.8 (0.1–1.4)
			P = 0.01	P = 0.02
Individuals without thyroid disease and euthyroid at baseline	Women	1,577	0.6 (0.4–0.9)	0.6 (0.4, 0.9)
			P<0.001	P<0.01
	Men	367	0.8 (0.1–1.4)	0.7 (0.02–1.3)
			P = 0.02	P = 0.04

*CI* confidence interval, *TSH* thyrotropin.

a Unadjusted model.

b Model adjusted for age, change in smoking and change in leisure time physical activity.

In addition, all the regression analyses were repeated with the predictor variable divided into quartiles, which showed essentially unchanged estimates (data not shown). No significant interaction with smoking status (yes/no) was found in any of the three models, and no significant interaction with region (used as a proxy for different levels of iodine intake) was observed in the regression of the association between TSH change and change in weight during follow-up (women, P for trend = 0.96; men, P for trend = 0.25). Moreover, we did not find any association between natural logarithm to creatinine-adjusted iodine excretion at baseline and change in weight in an age-adjusted, sex-stratified multiple linear regression model (data not shown).

We found no significant association between baseline free T4 level and weight change during 11-year follow-up among individuals without treatment for thyroid disease (women, P = 0.58; men, P = 0.42), and in persons with a TSH within the normal range at baseline (women, P = 0.44; men, P = 0.36). An inverse association was found between 11-year free T4 change and weight change in non-treated women (−0.21 kg change/(pmol/liter change) (95% CI, −0.33–−0.09), P = 0.008), but not significant in men (−0.16 kg change/(pmol/liter change) (95% CI, −0.37–0.05), P = 0.14). In persons with a TSH within the normal range at baseline similar results were found (women, −0.22 kg change/(pmol/liter change) (95% CI, −0.35–−0.10), P = 0.0005; men, −0.19 kg change/(pmol/liter change) (CI, −0.41–0.03), P = 0.09).

**Figure 2:**
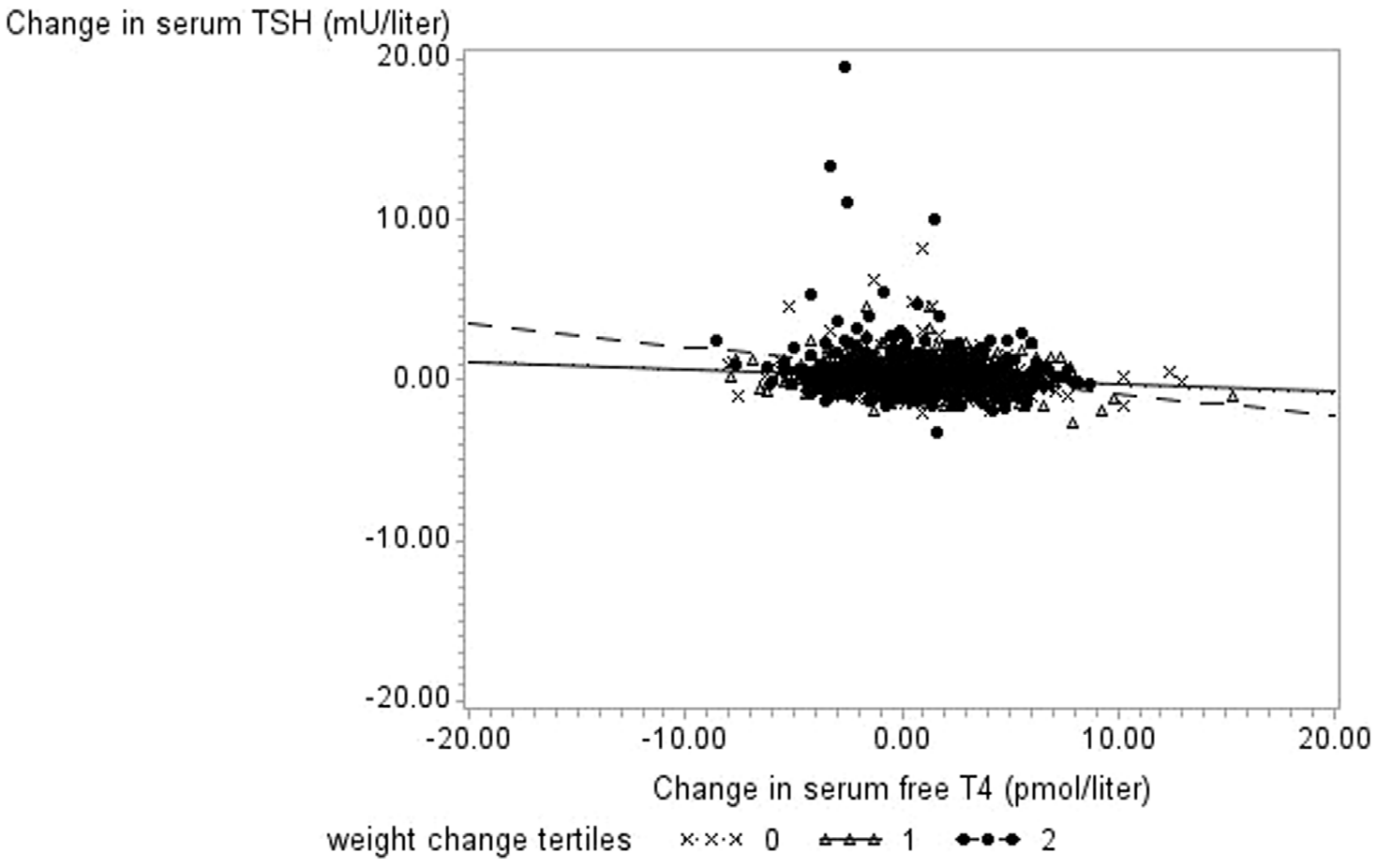
Unadjusted regression lines for weight change tertiles in women (n = 1,577). 11-year weight change was divided in three groups, a lower, middle and upper tertiles. In the adjusted regression model, regression coefficients (β) for the association between Δfree T4 and ΔTSH differed significantly in the three groups (P<0.0007 for the interaction between Δfree T4 and Δweight tertiles), and was estimated to: β_lower Δ weight tertile_ = −0.047 mU/L (CI, −0.096, −0.0018), β_middle Δ weight tertile_ = −0.049 mU/L (CI, −0.093, −0.005), and β_upper Δ weight tertile_ = −0.150 mU/L (CI, −0.191, −0.109). Note the lines of the lower and middle weight change tertiles are difficult to distinguish from each other. Adjustments were made for age group (categorical), change in smoking status, change in leisure time physical activity, and for the baseline value of the outcome.

As expected, we found an inverse age-adjusted association between TSH change and free T4 change (P<0.0001, data not shown). Regression coefficients of the three weight change tertiles differed significantly in linear regression analysis of the association between change in serum TSH concentration and free T4 change (P<0.001) (**Figure 2**). The slope of the regression line was more negative in persons in the upper weight change class (tertile), than in the lower and middle tertiles. Serum TSH decreased by −0.150 mU/L (CI, −0.191, −0.109) and by −0.047 mU/L (CI, −0.096, −0.0018) for every one unit (pmol/liter) increase in free T4, for persons with a weight change in the upper and lower tertiles, respectively.

Among the 4,649 persons participating in the DanThyr baseline study, 403 persons died and 72 emigrated before or during the 11-year follow-up. Comparison of these persons with the survivors who participated in the follow-up showed no difference between the groups concerning baseline values of serum TSH concentrations (P = 0.40), weight (P = 0.75), and BMI (P = 0.08), as previously reported [24].

## Discussion

In this community-based sample of 1,944 adults followed for 11 years, we found a statistically significant positive association between change in serum TSH concentrations over time and weight change. However, baseline TSH concentration was not a determinant of change in weight and baseline BMI level was not a determinant of change in TSH during follow-up. The relationship between change in TSH and free T4 change differed significantly between groups with different weight changes.

Several cross-sectional population studies found a significant positive association between serum TSH levels and BMI or body weight in individuals free of thyroid disease, in both sexes [13], [16], or in women only [15]. Also, in persons with TSH levels within the normal reference range, a significant association with body weight was confirmed [14], [18], though restricted to non-smoking subjects in one study [17]. Two studies could not confirm the association [30], [31]. Possible explanations for the lack of association found by Manji et al. [31] could be the small sample size (N = 400), the different clinical spectrum of the studied population (euthyroid hospital-referred patients with thyroid nodules or goiter), and the use of correlations without adjustment for confounders. Our result regarding the significant, positive association between change in serum TSH and change in body weight is in line with the results of the four existing longitudinal population studies [14], [17], [19], [20]. However, the association was restricted to women in the study by Gopinath et al. [19] and to non-smokers in the Tromsø study [17]. Regarding the observed lack of association between baseline TSH and weight change during follow-up, the same was observed by Fox et al. [14] and Svare et al. [20]. Svare et al. [20], contrary to our study, found a negative association between baseline BMI and change in serum TSH. Fox et al. [14] and Nyrnes et al. [17] did not investigate the association between baseline BMI and TSH change.

Fat tissue could affect thyroid hormones in a number of ways. First, leptin, an adipocyte-derived hormone, could affect thyroid hormone status through the stimulation of hypothalamic thyrotropin-releasing hormone (TRH) gene expression [32], [33] and influence thermogenesis [3]. However, as described in a recent review, the role of leptin seems to be restricted to energy-deficient states such as acute fasting and exercise, and evidence in humans is still scarce [34]. Second, the effect may be due to a change in deiodinase activity in central and/or peripheral tissue [2], [9]. Third, hormone resistance in obese subjects as a result of a T3 receptor decrease has been suggested [33]. Fourth, a partially bioinactive TSH protein in obese subjects has been speculated [33].

Our longitudinal results provide insight into the association between body weight and TSH found in cross-sectional observational studies. An association between weight and serum TSH seems to be present at all time points. However, the association we found does not necessarily reflect a causal relationship. Both TSH and weight might be modified by a third factor. The absence of a predictive value of TSH for weight change and of BMI for TSH change could suggest that this might be the case.

A modifying effect of tobacco smoking on the relationship between thyroid function and BMI in euthyroid subjects has been suggested [30]. Our analyses do not, however, support tobacco smoking as a modifying factor, as we found no significant interaction with change in smoking status in any of the models.

Previous studies of the TSH/free T4 relationship have shown an exponential decrease in TSH with progressive increase in serum T4 [35], [36]. Ercan-Fang et al. suggested [21], in a study of patients with hereditary pituitary resistance to thyroid hormones, that assessment of the slope of lnTSH as a function of free T4 was a useful measure of the abnormal thyroid hormone sensitivity. The exact mechanism behind the observed difference in the ΔTSH/Δ free T4-relation for the upper, middle and lower tertiles of weight change, can not be resolved on the basis of this study. Possible suggestions could however be a higher pituitary response to change in free T4 with increasing weight, a less biological active TSH molecule with increasing weight, or even more speculative, that the thyroid glands response is less sensitive to TSH with increasing weight.

Our cohort was exposed to a program of mandatory iodization of salt, resulting in an increased iodine urine excretion [24]. It may be speculated that the increased iodine intake could affect the studied association. To the best of our knowledge, no studies have described an association between iodine intake and weight, which speaks against iodine as a possible confounder of the studied associations. Moreover, the absence of interaction with region (as a proxy for different levels of iodine intake) in the adjusted model of weight change and TSH change and the fact that creatinine-adjusted iodine excretion was not associated with change in weight imply that the iodization program was unlikely to have had a major effect on the studied association.

The strengths of our study are the longitudinal population-based design, ensuring the temporality of studied associations; the long-term follow-up; the objective measurement of exposures and outcomes; the measurement of free T4 beside TSH at baseline and follow-up; and the detailed information on covariates. The limitations could be residual confounding from unmeasured confounders such as diet or non-thyroidal illness; and selection bias as a consequence of the relatively low participation rate of 59.1%. Furthermore, TSH is a measurement known to have a large within individual variation and analytical variation [37], and the fact that the determination of TSH relied on a single measurement at baseline and at follow-up could bias the values of change in TSH during follow-up. This would, however, only lead to a nondifferential misclassification. Repeated measurements of TSH and weight in each individual during the follow-up period would more precisely outline the shape of their relationship with time [38].

## Conclusion

In conclusion, TSH levels are not a determinant of future weight changes and BMI is not a determinant of TSH changes, but changes in TSH levels seems to be positively associated with weight change. It remains to be proven whether body weight and serum TSH are causally connected.
